# Antimicrobial Resistance in *Enterobacterales* and Its Contribution to Sepsis in Sub-saharan Africa

**DOI:** 10.3389/fmed.2021.615649

**Published:** 2021-01-26

**Authors:** Kathleen Tompkins, Jonathan J. Juliano, David van Duin

**Affiliations:** ^1^Division of Global Health and Infectious Diseases, University of North Carolina, Chapel Hill, NC, United States; ^2^Department of Epidemiology, Gillings School of Global Public Health, University of North Carolina, Chapel Hill, NC, United States; ^3^Curriculum in Genetics and Molecular Biology, School of Medicine, University of North Carolina, Chapel Hill, NC, United States

**Keywords:** resistance, *Klebsiella pneumoniae*, *E. coli*, sepsis, Sub-Sahara Africa

## Abstract

Antibiotic resistant *Enterobacterales* (formerly *Enterobactereaceae*) are a growing threat to Sub-Saharan Africa. Genes causing antibiotic resistance are easily spread between the environment and humans and infections due to drug resistant organisms contribute to sepsis mortality via delayed time to appropriate antimicrobial therapy. Additionally, second or third-line antibiotics are often not available or are prohibitively expensive in resource-constrained settings leading to limited treatment options. Lack of access to water and sanitation facilities, unregulated use of antibiotics, and malnutrition are contributors to high rates of antibiotic resistance in the region. Improvements in the monitoring of drug resistant infections and antibiotic stewardship are needed to preserve the efficacy of antibiotics for the future.

## Introduction

Globally, sepsis is estimated to cause an excess of 5 million deaths annually with low and middle income countries disproportionately affected (1). Sepsis is a significant problem in Sub-Saharan Africa (SSA) where mortality rates can be as high as 43% (2–4). While this high mortality is in part due to a lack of critical care resources being available in much of SSA (5), drug resistant pathogens play an important role in the increased mortality due to sepsis in this region.

Antimicrobial resistance (AMR) is a significant threat to human health that hampers our ability to treat a wide range of bacterial, parasitic, fungal, and viral infections (6). Antimicrobial resistance is recognized by the World Health Organization (WHO) as an increasingly serious threat to global public health that requires action across all government sectors and society (7). Although much of the research into AMR to date has been conducted in high-income countries (8), low and middle income countries (LMICs) bear a growing burden of AMR (9). Countries in SSA, in particular, are understudied in terms of rates and excess mortality due to AMR infections (10), yet experience a significant burden of disease (11, 12) and are expected to bear a disproportionate mortality burden (13). Additionally, parts of SSA have comparatively high rates of people living with HIV, which has been shown to increase the risk of invasive infection and subsequent sepsis (14, 15).

It is well-established that there are worse outcomes with drug-resistant infections in high-income countries (16–18). These poor outcomes can be exacerbated in low income regions where drug susceptibility testing can be delayed or absent and second or third-line antibiotics are either unavailable or prohibitively expensive (19). Additionally, AMR has been forecast to increase the number of people living in extreme poverty globally (20), exacerbated by global inequities and poor access to healthcare.

*Enterobacterales* (formerly *Enterobacteriaceae*) are of particular concern in SSA given their ability to rapidly colonize and spread (21–23) and the limited treatment options available for drug-resistant *Enterobacterales* in resource-constrained settings (24–26). Both the WHO and the CDC recognize drug-resistant *Enterobacterales* as extremely concerning pathogens (27, 28). This review will focus on the rates and causes of AMR in *Enterobacterales* in SSA and the subsequent contribution to sepsis and mortality in the region. A limitation of this review is that more data are needed from future studies to more precisely estimate the rates and mechanisms of resistance in specific bacterial species, and the patient outcomes associated with infections caused by specific multidrug-resistant organisms.

## Genetic Diversity of AMR in SSA

Antimicrobial resistance can occur through a variety of mechanisms. For *Enterobacterales*, resistance to the beta-lactam class of antibiotics is particularly concerning as beta-lactams are the cornerstone of treatment for these infections (29–31). Resistance to beta-lactam antibiotics can occur via efflux pumps, altered penicillin binding sites, and beta-lactamases which cleave the beta-lactam ring and inactivate the target antibiotic (32), with beta-lactamases being the primary means of resistance for cephalosporins and carbapenems. Development of resistance occurs through new mutations or upregulation and expression of existing genes, *AmpC* being the most common occurrence of the later (33). Spread can then occur through clonal dissemination, horizontal gene transfer via plasmids, or translocation of resistance genes between mobile genetic elements (32, 34).

As AMR genes have spread worldwide they are now found throughout Africa in varying rates. The beta-lactamase genes *bla*_*CTX*−*M*_ are responsible for much of the spread for ESBL-Enterobacterales (ESBL-E) worldwide (35–37). *bla*_*CTX*−*M*_ are most commonly transferred via plasmids (38), which allow for the spread of multiple resistance genes at one time (39). In Africa, rates of ESBL-E are increasing, largely as a result of CTX-M genes. A review of AMR isolates in East Africa found high rates of a variety of AMR genes, with the *bla*_*CTX*−*M*_ genes being the most common etiology of ESBL-E infection, found in 45.7% of isolates (40). A study in Ethiopia showed high rates of ESBL infections among gram negative isolates with 95% of those carrying CTX-M genes (41). High rates of CTX-M have similarly been found in studies from Nigeria (42), Tanzania (43, 44), Malawi (45, 46), and Ghana (47). Other ESBL-encoding genes including *bla*_*TEM*_*, bla*_*OXA*_, and *bla*_*SHV*_ are also found through the region (40, 46).

In addition to ESBL-producing organisms, carbapenemase-producing organisms are a growing threat to the region. The carbapenemase gene KPC is the most prevalent carbapenemase in the United States (48), but is less frequently found in Africa (49). In contrast, the carbapenemase genes *bla*_*IMP*_*, bla*_*VIM*_, and *bla*_*OXA*_ appear to be more common in Africa (40, 50). For carbapenemase genes, it remains unclear if clonal expansion or horizontal transfer via plasmids are the primary means of dissemination (51), however they have been shown to spread easily and rapidly in hospitals and other healthcare settings (52). This is of particular concern in SSA where rapid diagnosis is not always possible and strict hygiene and infection control measures can be difficult to achieve (53, 54).

## Burden of Intestinal AMR Carriage in SSA

There is substantially less data characterizing the rates of AMR carriage and infection in SSA when compared to North America and Europe. Studies that have been done are primarily from urban areas, despite 60% of the population of SSA living in rural regions (55). Studies can be broadly categorized as those that determine rates of AMR carriage in community-dwelling (asymptomatic) individuals and those that are hospital-based and determine rates in patients with active infection.

Although infection rates are an obvious and important source of information to assess the contribution of AMR to sepsis in SSA, carriage rates are equally important, as intestinal colonization with AMR pathogens precedes and predicts subsequent infection. The intestinal microbiome is a well-described and important reservoir for AMR bacteria that may cause subsequent infections (56–58) and the likelihood of invasive AMR infections is dramatically increased in people who are colonized (59, 60).

Rates of intestinal AMR carriage can be studied in a variety of ways, which can make comparison between studies difficult, however rate of ESBL-E carriage is a common method. In SSA, rates of ESBL-E carriage vary widely by the region and specific population being studied, from 5 to 59%. Carriage rates from a number of studies and their populations are summarized in Table 1. Notably, many of these studies include individuals with little to no healthcare exposure or antibiotic use, suggesting there is significant community spread.

**Table 1:** Studies evaluating rates of ESBL-E carriage in SSA.

**References**	**Country**	**Population**	**Percent with ESBL intestinal carriage (%)**
Sanneh et al. (61)	The Gambia	Food handlers	5
Farra et al. (62)	Central African Republic	Children (age 0–59 months)	59
Mshana et al. (63)	Tanzania	Any age	16.5
Chereau et al. (64)	Madagascar	Pregnant women at time of delivery	18.5
Ribeiro et al. (65)	Angola	Healthy community dwellers	22.2
Albrechtova et al. (66)	Kenya	Nomadic pastoralists	17
Tellevik et al. (67)	Tanzania	Healthy children <2 y/o	11.6
Moremi et al. (68)	Tanzania	Street children	31.8
Chirindze et al. (69)	Mozambique	University students	20
Fortini et al. (70)	Nigeria	Healthy pregnant women on day of admission	31.7
Ruppé et al. (71)	Senegal	Children in remote village	10
Lonchel et al. (72)	Cameroon	Student volunteers	6.7
Ouchar Mahamat et al. (73)	Chad	Healthy community volunteers	38

## Rates of AMR Infections in Hospitalized Patients

Asymptomatic carriage can be a precursor to disease, and, while there are fewer studies from SSA than from other regions, hospital infections with AMR pathogens is an important contributor to disease in this region. This is particularly relevant for bloodstream infections (BSI), as BSI remain a leading cause of death of both adults and children in the region (74). Ceftriaxone is often used empirically for sepsis in the region due to the relative ease of dosing and low cost. Thus, ceftriaxone resistance is particularly worrisome. The MERINO study showed that carbapenems are the preferred treatment option for invasive infections caused by ceftriaxone-resistant enteric bacteria (75). Unfortunately, carbapenems are not available to many patients with sepsis in SSA.

A recent meta-analysis of bloodstream infections in SSA found an overall prevalence rate of third generation cephalosporin resistance of 18.4% for *Escherichia coli* isolates and 54.5% for *Klebsiella* isolates (19). Some studies suggest the rates of drug resistance are increasing dramatically in the region. For example, a study of BSI from a large referral hospital in Malawi showed an increase in ESBL resistance from 0.7 to 30.3% of *E. coli* isolates and from 11.8 to 90.5% in *Klebsiella* spp. isolates between 1998 and 2016 (76). Similarly, they found methicillin resistance of *Staph aureus* isolates increased from 7.7 to 18.4% over the same period. More data are needed from future studies to more precisely estimate the rates and mechanisms of resistance in specific bacterial species, and the patient outcomes associated with infections caused by specific multidrug-resistant organisms.

## Outcomes of AMR Infection in Hospitalized Patients

The dramatic rise in AMR detection is associated with worse outcomes. A study in pediatric patients at a hospital in Senegal showed that AMR carriage is associated with delayed time to appropriate antibiotics and a substantial increase in mortality from 15.4 to 54.8% in patients with ESBL-producing *Enterobacterales* BSI compared to susceptible strains (77). This was confirmed by another study from Tanzania which found AMR to be an independent risk factor for mortality in both children and adults with BSI, with mortality rates increasing from 13.7 to 48.4% when organisms were multidrug resistant compared to those that were susceptible (78). Similarly, a study of adult patients in Ethiopia showed significant increase in mortality with BSI if the isolate was resistant to third generation cephalosporins, with all patients with resistant infection in that study having died (79).

Aside from BSI, resistance presents a challenge to other infections as well. Clinical isolates from multiple sources including wound, urine, blood, and sputum at a large Rwandan hospital showed 75.9% of isolates were resistant to ceftriaxone, a third generation cephalosporin (80). A study of clinical isolates growing *Klebsiella pneumoniae* from Cote d'Ivoire likewise showed high prevalence of ESBL resistance, at 84% of all isolates and 94% of pediatric isolates (81). These findings have implications for the treatment of urinary tract infections, wound infections, and surgical infections, among others.

Although many patients present from the community with AMR infections, hospital acquisition is a common means of becoming infected or colonized with AMR pathogens and can occur soon after admission. A study from Rwanda looking at ESBL-E acquisition during hospitalization found that overall carriage rates in patients increased from 49.7% on admission to 64.6% on discharge, however this number was as high as 93% on discharge from the pediatric unit (82). Another study of neonates in Kenya showed that 55% acquired ESBL carriage during hospitalization (83).

## Drivers of AMR in SSA

AMR is driven by multiple factors, however overconsumption with unnecessary or inappropriate use of antibiotics is a significant contributor (84). Consumption of antibiotics varies widely between regions, however, as many as half of all antibiotics in developing countries being used inappropriately (85). A recent WHO report from a limited number of countries in Africa shows rates of antibiotic consumption that range from 4.4 to 27.3 daily doses per day per 1,000 inhabitants (86). Notably, this only includes antibiotics dispensed through regulated agencies, not unregulated or non-prescription sales. Dispending antibiotics without a prescription is a common occurrence in much of Africa. In one region of Tanzania, over 88% of antibiotic prescription were found to be irrational with 76% occurring without a prescription (87) and in Cameroon 47% of antibiotics dispensed at pharmacies were without a prescription (88). This lack of regulation and consumption of inappropriate and non-prescription antibiotics is a contributor to the spread of drug resistance in the region (89–92).

Human consumption of antibiotics is only one driver of AMR. Livestock and veterinary overuse of antibiotics for commercial farming are a large contributor of AMR worldwide (93, 94) and this holds true in SSA as well (95). The idea that humans, animals, and the environment are connected and all contribute to the health of society is termed a “One Health” approach (96). Antibiotics used in farming practices are discharged into the environmental through animal waste where they contribute to the “resistome” of the environment and can lead to human colonization or infection with AMR pathogens, raising the overall AMR burden in communities (97). Antibiotic use in commercial animal farming has largely focused on Europe, North America, and certain middle income countries including China, Brazil, and India where use rates are high (98), however high rates of antibiotic use and subsequent AMR have been found in SSA ranging from 74 to 100% of farms (99), with little to no surveillance systems in place on national levels to ensure accurate tracking of use (100). Additionally, antibiotic use for livestock worldwide is expected to rise significantly in the coming years, including in SSA, with an estimated 67% increase to 105,000 tons by 2030 (93).

The multidisciplinary nature of AMR was highlighted by a recent study showing that the spread of AMR genes (as opposed to selective bacterial pressure) may be the dominant factor contributing to high rates of AMR in the region (101). To this end, improving access to safe drinking water and sanitation facilities (102) are urgently needed to help limit AMR (103).

In addition to the above, malnutrition may also contribute to AMR in SSA, particularly in the pediatric population. It is well-established that malnutrition places individuals at increased risk for infection (104) and thus is may facilitate the spread of AMR genes (105). Additionally, malnutrition may place individuals at greater risk specifically for drug-resistant infections. In Senegal, malnutrition was associated with twice the risk for contracting an ESBL-BSI compared to a susceptible BSI (77). Similarly, another study found high rates of multidrug resistant urinary tract infections in malnourished children in Tanzania (106).

Africa has the highest number of people living with HIV (PLWH) in the world. There is growing evidence that HIV may increase the carriage rates of AMR bacteria in the GI tract (107), a precursor to invasive infection. Two studies of pediatric patients hospitalized in South Africa with BSI found that HIV infection was associated with increased likelihood of having a resistant isolate and a corresponding increase in sepsis-related mortality (108, 109). Similarly, a study of pediatric bacteremia in Tanzania found children living with HIV were more likely to receive inappropriate initial antimicrobial therapy due to drug resistance and subsequently had a higher risk of mortality compared with their HIV negative counterparts (110).

## Global Importance of AMR

Although this review focuses on AMR and its contribution to sepsis in SSA, drug resistance is a global problem that extends beyond national borders. As travel and immigration lead to increasing globalization, AMR genes can quickly spread worldwide (23). A recent meta-analysis found that a quarter of migrants to Europe were colonized with multi-drug resistant organisms, with the rate being higher in asylum seekers (21). Similarly, a study of Eritrean migrants in Europe showed high rates of gram negative and polymicrobial carriage with AMR genes detected (111). International travel is also a means of dissemination, with 24% of returning Swedish travelers carrying CTX-M-15 (112) and a meta-analysis showing overall acquisition of ESBL organisms among travelers to Africa to be over 15%, with wide variation between regions and higher rates in those who experienced travelers diarrhea or used fluoroquinolone antibiotics while abroad (113). In addition, acquisition is not confined to the traveler, as another study showed the probability of transmitting AMR genes to another household member after return was 12% (114), indicating the potential for dissemination to the surrounding community.

## Conclusions

Antimicrobial resistance is growing as a worldwide threat and contributes to sepsis-associated mortality, with alarming rates in SSA. Moving forward, a global coordinated response is needed to halt the spread of AMR and improve outcomes for patients with sepsis due to these organisms in SSA. To this end, the World Health Organization developed the Global Action Plan Against Antimicrobial Resistance in 2015 to create a framework for member states to focus research and resources on AMR while attempting to reduce the need for antibiotics through improved infection prevention and sanitation (115). That same year, the WHO launched the Global Antimicrobial Resistance Surveillance System (GLASS) to uniformly track rates of AMR worldwide and estimate the attributable mortality due to AMR (116). As AMR continues to pose a threat to human health across the world it is clear that a multi-pronged approach is needed to halt the spread. This will require cooperation between countries and across disciplines and is imperative to ensure the continued efficacy of antimicrobials.

## Author Contributions

KT, JJ, and DD searched and reviewed the literature, summarized the data, and reviewed and edited the final content. KT wrote the first draft of the paper. All authors contributed to the article and approved the submitted version.

## Conflict of Interest

DD has served as a consultant for Achaogen, Allergan, Shionogi, Tetraphase, Pfizer, Merck, T2 Biosystems, Karius, Utility, Entasis, Qpex, and Roche. The remaining authors declare that the research was conducted in the absence of any commercial or financial relationships that could be construed as a potential conflict of interest.
